# Imaging Off‐Resonance Nanomechanical Motion as Modal Superposition

**DOI:** 10.1002/advs.202005041

**Published:** 2021-05-19

**Authors:** Joshoua Condicion Esmenda, Myrron Albert Callera Aguila, Jyh‐Yang Wang, Teik‐Hui Lee, Chi‐Yuan Yang, Kung‐Hsuan Lin, Kuei‐Shu Chang‐Liao, Nadav Katz, Sergey Kafanov, Yuri A. Pashkin, Chii‐Dong Chen

**Affiliations:** ^1^ National Tsing Hua University Hsinchu 30013 Taiwan; ^2^ Nano Science and Technology Program, Taiwan International Graduate Program, Academia Sinica National Taiwan University and National Tsing Hua University, Institute of Physics, Academia Sinica Nangang Taipei 11529 Taiwan; ^3^ Institute of Physics Academia Sinica Nangang Taipei 11529 Taiwan; ^4^ National Tsing Hua University Hsinchu 30013 Taiwan; ^5^ Racah Institute of Physics Hebrew University Jerusalem 91904 Israel; ^6^ Department of Physics Lancaster University Lancaster LA1 4YB United Kingdom; ^7^ Institute of Physics Academia Sinica Nangang Taipei 11529 Taiwan

**Keywords:** modal superposition, nanomechanical motion, off‐resonance

## Abstract

Observation of resonance modes is the most straightforward way of studying mechanical oscillations because these modes have maximum response to stimuli. However, a deeper understanding of mechanical motion can be obtained by also looking at modal responses at frequencies in between resonances. Here, an imaging of the modal responses for a nanomechanical drum driven off resonance is presented. By using the frequency modal analysis, these shapes are described as a superposition of resonance modes. It is found that the spatial distribution of the oscillating component of the driving force, which is affected by both the shape of the actuating electrode and inherent device properties such as asymmetry and initial slack, greatly influences the modal weight or participation. This modal superposition analysis elucidates the dynamics of any nanomechanical system through modal weights. This aids in optimizing mode‐specific designs for force sensing and integration with other systems.

## Graphical abstract

Nanomechanical motion in between resonances are imaged and then described as superposition of resonance modes. The modal weight behavior allows for the elucidation of the dynamics of any nanomechanical system such as the spatial distribution of the oscillating driving force. This analysis demonstrates that spatial imaging of mechanical motion is more than just a visualization tool.

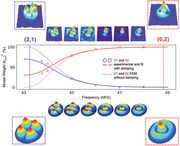

## 1 Introduction

Mechanical vibrations of a structure, regardless of its geometry and type of material, can be fundamentally described by a combination of its natural resonance modes, called eigenmodes, and a form of the driving force.^[^ ^1^ ^]^ A resonance mode is defined as a pattern of motion in which the system or its part moves in a periodic oscillation with a maximal amplitude at a characteristic frequency. The spatial distribution of motion is determined by the inertial and elastic properties of the oscillating body, its shape, and the boundary conditions imposed on it. If either the material properties or the boundary conditions of the structure change, the resonance modes change accordingly.^[^ ^2^ ^]^ A number of groups have included spatial imaging of mechanical mode shapes,^[^ ^3^, ^4^, ^5^, ^6^, ^7^, ^8^, ^9^, ^10^ ^]^ and while all of them are focused on resonances, the full potential of mode imaging has yet to be explored. While the resonance modes are characteristic of the inherent properties of the structure, their visualization requires the application of a driving force, which provides an external stimulus by which one can excite these resonance modes. An example of such force is the time‐varying electrostatic force between the two plates, one of which is flexible, of a parallel‐plate capacitor when an alternating voltage is applied across the plates.^[^ ^11^ ^]^ Interestingly, this geometry is typically used in studies of 2D nanomechanical resonators (NMRs), which found their niche as sensitive tools for measurements of various properties such as electrical conductance, thermal conductance, mass, radiation power, and many more.^[^ ^2^, ^11^, ^12^, ^13^ ^]^ Unique mechanical properties of 2D materials, which include low mass, high flexibility, and high tensile strength among others, allow them to have a large amplitude of flexural motion, making the NMR a viable object for coupling to other systems^[^ ^14^, ^15^ ^]^ such as superconducting cavities.^[^ ^16^, ^17^, ^18^ ^]^ It is fundamentally important to know then how the shapes of the actuating electrodes and resonance modes affect each other. Observing the off‐resonance motion and how the modal shapes transition from one to another may provide insights into understanding this relationship.

In this paper, we explore the nature of the driving force in the system through the imaging of off‐resonance motion. We investigate the response shapes, for driving frequencies both at resonance and off resonance, of the 2D mechanical plate drums made from niobium diselenide (NbSe_2_) flakes using a Fabry‐Perot laser interferometer. ^[^ ^19^ ^]^ By observing the off‐resonance response shapes, we see transitions from one resonance mode to another. Using frequency modal analysis,^[^ ^21^, ^22^ ^]^ we determine how the resonance modes of the system participate in vibrations, with some being more prominent than the others, as the driving frequency changes. A consequence of this is the demonstration of how the resonant modes depend on the spatial distribution of oscillating component of the driving force. This force spatial distribution then allows us to infer the inherent asymmetry and the initial slack of the system.

## 2 Results

### 2.1 Description and Characterization of the Device

**Figure 1:**
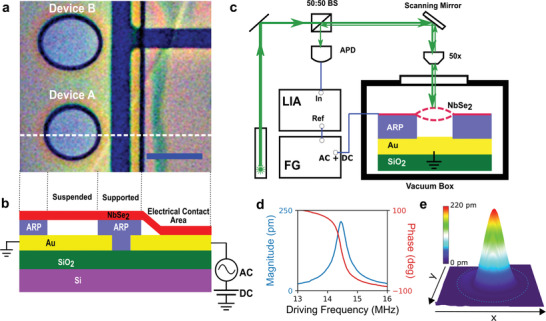
Characterization of NbSe_2_ mechanical drums using laser interferometry. a) Optical image of the two mechanical drums (scale bar is 7 µm). b) A schematic cross‐section of the mechanical drums device. NbSe_2_ flake (red) is placed on top of a patterned 295 nm thick AR‐P electron beam resist (blue), which serves as a spacer. The flake is estimated to be about 55 nm thick using FEM simulations. There are two types of drumhead patterns, namely, a circle (device A) with a diameter of 7 µm, and an ellipse (device B) with minor and major axes lengths of 7 and 8 µm, respectively. In a couple of microns away from the drumhead patterns, there is a larger rectangular opening on the AR‐P resist spacer, where the flake is made to have electrical contacts with the gold bottom electrodes (in yellow). c) The sample is contained inside a vacuum box with a pressure of about 10^−7^ mbar. Detection of motion of the mechanical drums is done by focusing a continuous wave green (532 nm) laser on the drumhead and recording the interfering reflections from the flake and the gold electrode underneath using an avalanche photodetector (APD). A scanning mirror is used to move the laser spot across the drumhead to record spatially resolved responses of the drums. The beam splitter (BS) is used to direct the reflected signal to the APD. A lock‐in amplifier (LIA) then records the data from the APD while referenced by a function generator (FG). The drums are driven with a combination of AC and DC from a function generator. d) Frequency response (magnitude and phase) of the fundamental resonance mode of device A. e) Corresponding 3D plot at resonance of the magnitude as a function of *x* and *y* of device A. The *z* units are in pm and the spatial scanning range is about 12 µm × 12 µm. The magnitude of the driving signal is 250 mVpp with a DC of 4 V.

NbSe_2_ mechanical drums are fabricated through the transfer of exfoliated flakes onto a silicon chip with pre‐patterned gold electrodes and AR‐P (AllResist Positive) resist. The latter acts as a spacer for the drum (details of the fabrication are described in the Section 5 and Supporting Information). **Figure** **1**a shows an optical image of a sample containing a circular drum (device A), and an elliptical drum (device B). Figure 1b shows the schematic cross‐section of the chip along the white dashed line shown in Figure 1a. The motion of the mechanical drum is then detected using a laser interferometry technique as illustrated in Figure 1c (details of the mechanical detection and actuation are described in the Section 5). Figure 1d shows the magnitude and phase response at the fundamental resonance mode, which corresponds to a simple harmonic oscillator. Figure 1e is a 3D plot of the spatially resolved frequency response mapping of the fundamental mode. The setup maps the frequency dependence of the mechanical displacement, *Z*(*x*, *y*, ω_d_), where *x* and *y* are the mapping coordinates with the center of the drum as the origin, to the corresponding photodetector voltage signal *V*(*x*, *y*, ω_d_), where ω_d_ is the driving frequency. When ω_d_ is chosen to be the modal resonance frequency ω_*mn*_, where *m* and *n* refers to the number of nodal diameters and the number of nodal circles, respectively, for a clamped circular plate (the mode notation used is (*m*, *n*)), *V*(*x*, *y*, ω_d_) reflects the resonance mode shape *Z*_*mn*_(*x*, *y*). When ω_d_ is off‐resonance, *Z*(*x*, *y*, ω_d_) is the shape of the off‐resonance response. The conversion of *V*(*x*, *y*, ω_d_) into *Z*(*x*, *y*, ω_d_) was done by retrieving the responsivity of the device through the differentiation of the reflectivity with respect to spacer thickness. The multilayer interference approach for obtaining the total reflectivity of the device is fully explored in a separate article.^[^ ^23^ ^]^

### 2.2 Spatial Response Mapping

In order to visualize the response shapes, a system response to the drive at various frequencies is first measured for both devices at certain spots of their respective drumheads. From the knowledge of the theoretical mode shapes of a circular plate,^[^ ^24^, ^25^, ^26^ ^]^ we could predict the frequencies of the resonance modes that are being driven (shown in the Supporting Information). We also know that the lowest six modes are (0,1), (1,1)a, (1,1)b, (2,1)a, (2,1)b, and (0,2) in order of increasing frequency value, with the assumption of asymmetry. The a and b in the notation are used to designate the bifurcation of the circular modes into two elliptical modes due to the introduction of eccentricity.^[^ ^27^ ^]^

**Figure 2:**
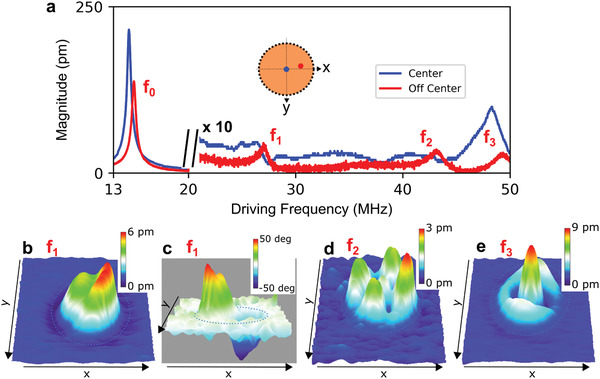
Resonant mode mapping of device A. a) Response spectra retrieved at two different locations on the drumhead. For clarity, the magnitude of the spectra was scaled to 10× above 20 MHz of the driving frequency. The spectrum taken at the drum center (shown in blue) has two pronounced peaks: the fundamental frequency at 14.46 MHz and a higher order resonance at 48.22 MHz. The off‐center spectrum (red) has four peaks labeled *f*_0_ (at 14.55 MHz) through *f*_3_. The inset shows location of the corresponding laser spot on the drumhead where the frequency response spectra are retrieved. b–e) Spatially resolved mapping measurements with the same *x* and *y* directions as in Figure 1e and with a step size of ≈189 nm in both *x* and *y*, with 64 steps in each direction. b) Magnitude of *f*_1_ (26.81 MHz) with *z* scale in pm. c) The corresponding phase mapping of *f*_1_ with scale in degrees is used to show that the two sections of the drum move in opposite directions. d) Magnitude of *f*_2_ (43.25 MHz) with *z* scale in pm. e) Magnitude of *f*_3_ (48.83 MHz) with *z* scale in pm.

First, we investigate the response of device A. In **Figure** **2**a, two frequency response spectra are taken at different spots of the drumhead; one at the center and the other about halfway to the edge. The spectrum taken at the center has two peaks. This is indicative of the resonant modes that do not have nodal diameters passing through the center. This description fits the fundamental mode (0,1), whose spatially resolved mapping is shown already in Figure 1e, and the (0,2) mode having a higher frequency. Now, to find the other resonance modes, we look at the spectrum taken off the center. It is important to note here that it may take several trials to carefully choose a spot that shows resonant peaks as each drum has its own nodal orientation. In the second spectrum, we identify four peaks, with frequencies labeled *f*_0_ – *f*_3_, and with *f*_0_ having a *Q* factor of 26, and the higher modes having *Q* factors of 15, 18, and 21 for *f*_1_, *f*_2_, and *f*_3_, respectively. The values of *f*_0_ and *f*_3_ are very close to the peak frequency values identified in the first spectrum, which indicates that the same modes, (0,1) and (0,2), are excited. This is indeed true as demonstrated in Figure 2e, where the mapping at *f*_3_ is shown and it resembles the (0,2) mode shape. The slight frequency shift between the two spectra is presumably due to laser heating causing a temperature gradient across the drumhead, which in turn causes the material to expand, inducing changes in the tension.^[^ ^6^, ^28^ ^]^ We do the same for *f*_1_ and *f*_2_. Figure 2b,c presents the spatial mapping at frequency *f*_1_ and the shape resembles (1,1) mode. In Figure 2d, the spatial mapping at frequency *f*_2_ is shown and the shape resembles the (2,1) mode. However, the corresponding modal pairs of the *f*_1_ and *f*_2_ were difficult to find. A possible explanation could be their non‐favorability given the intrinsic asymmetry of the system. Now that we have mapped the response shapes at the resonances of the drum, we proceed to map the response shapes at frequencies between these resonances.

### 2.3 Modal Analysis

#### 2.3.1 Modal Weights from Experiment

**Figure 3:**
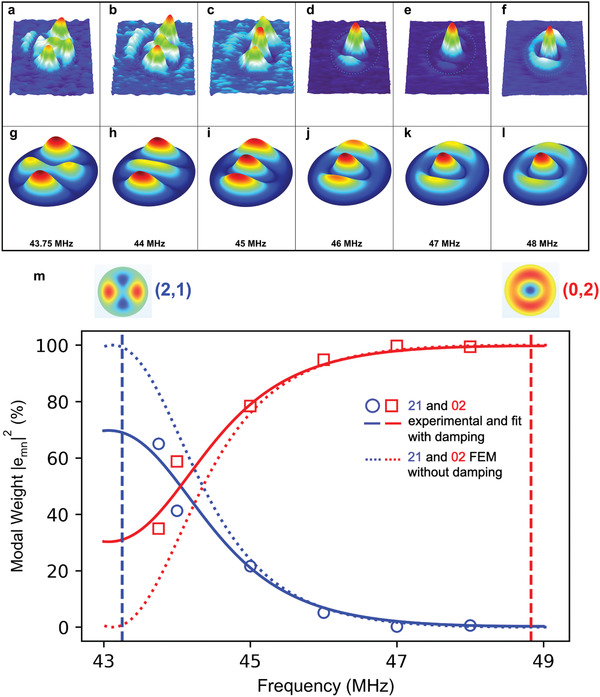
Off‐resonance modes of device A. a–f) Magnitude mapping at driving frequencies between the (2,1) mode through the (0,2) mode (The respective maximum amplitudes are as follows: 2.5, 2.2, 2.5, 3.5, 5.7, and 8.2 pm). g–l) Corresponding simulated magnitude mapping. All mappings have the same (*x*,*y*) directions as in Figure 1e. m) Modal weight for (2,1) and (0,2) modes as a function of the driving frequency: the circle and square symbols are obtained from the experiment using Equation (2), the dotted lines are from the FEM simulations, and the solid lines represent the fitting using Equation (6). The vertical dashed lines indicate the resonance frequencies of the (2,1) and (0,2) modes with the corresponding modal shapes shown above.

**Figure** **3**a–f shows the experimental off‐resonance shapes *Z*(*x*, *y*, ω_d_) of the circular drum, where $\frac{\omega_{d}}{2\pi }$ is between *f*_2_ ((2,1) mode) and *f*_3_ ((0,2) mode). From these, we can observe a transition of *Z*(*x*, *y*, ω_d_) from *Z*_21_(*x*, *y*) to *Z*_02_(*x*, *y*). As *Z*(*x*, *y*, ω_d_) evolves from one mapped mode shape to a neighboring one, a natural thought would be to describe *Z*(*x*, *y*, ω_d_) as a superposition of *Z*_*mn*_(*x*, *y*):

(1) $$Z\left(x,y,\omega_{d}\right)=\sum_{mn}Z_{mn}\left(x,y\right)e_{mn}\left(\omega_{d}\right)$$

where *e*_*mn*_(ω_d_) is the frequency‐dependent weights of the corresponding resonance mode, *Z*_*mn*_. Note that both $Z(x,y,\omega_{d})$ and $Z_{mn}\left(x,y\right)$ are normalized so that $\int_{A}{\left|Z\left(x,y\right)\right|}^{2}dxdy=1$ for the drum area *A*. Given $Z(x,y,\omega_{d})$ and $Z_{mn}\left(x,y\right)$, *e*_*mn*_(ω_d_) is determined by the following integral:

(2) $$e_{mn}\left(\omega_{d}\right)=\int_{A}Z_{mn}^{*}\left(x,y\right)Z\left(x,y,\omega_{d}\right)dx\;dy$$

**Figure 4:**
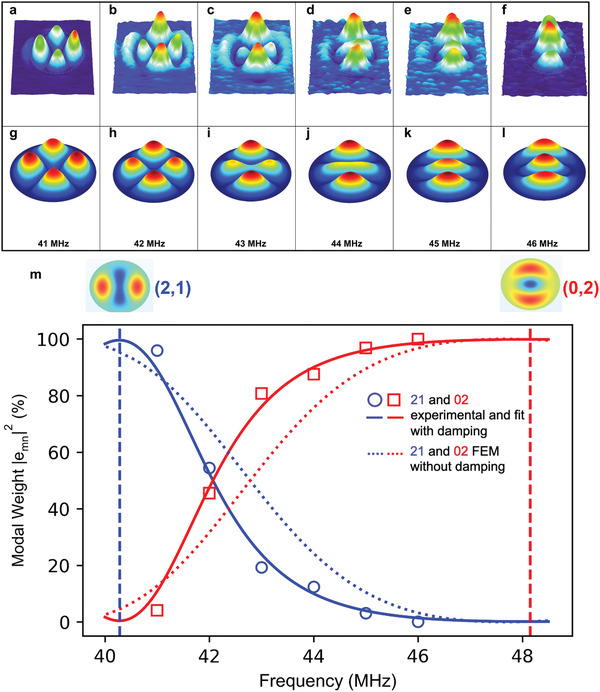
Off‐resonance modes of device B. a–f) Magnitude mapping at driving frequencies between the (2,1) mode through the (0,2) mode (The respective maximum amplitudes are as follows: 10, 3.5, 2.5, 2.2, 2.5, and 3.1 pm). g–l) Corresponding simulated magnitude mapping. All mappings have the same (*x*,*y*) directions as in Figure 1e. m) Modal weight for (2,1) and (0,2) modes as a function of the driving frequency: the circle and square symbols are obtained from the experiment using Equation (2), the dotted lines are from the FEM simulations, and the solid lines represent the fitting using Equation (6). The vertical dashed lines indicate the resonance frequencies of the (2,1) and (0,2) modes with the corresponding modal shapes shown above.

The circle and square symbols from Figure 3m show the dependence of the experimental modal weight, |*e*_*mn*_(ω_d_)|^2^, on the drive frequency, in percentage for the (2,1) and (0,2) modes. It is important to note here that the contributions of the other modes are not shown because their modal weights are almost zero (detailed results for all six modes are included in Supporting Information). A similar analysis was also performed for the elliptical drum and the off‐resonance shapes and corresponding experimental modal weights are shown in **Figure** **4**a–f and circle and square symbols in Figure 4m, respectively. In both drums, a gradual transition of the modal weight dominance from (2,1) to (0,2) is observed. To confirm this trend, we used finite element method (FEM) simulations.

#### 2.3.2 Modal Analysis Without Damping Using FEM Simulation

Figure 3g–l presents the corresponding mechanical displacement *Z*(*x*, *y*, ω_d_) obtained from FEM simulations using COMSOL neglecting damping parameters. The dotted lines in Figure 3m are the simulation results and show a reasonable agreement with the experimental data. We see that in the absence of damping, participation at the resonance frequencies is equal to 100%, which is not always the case in the experiment. From here we can see the transition of the modal weight dominance from (2,1) mode to (0,2). The FEM results show that at 44 MHz, the contributions are  45% and  55% for the (2,1) and (0,2) modes, respectively. In contrast, at 48 MHz, the response shape is overwhelmingly dominated by the (0,2) mode, while (2,1) mode contributes  0.07% only.

We have done the same analysis for the elliptical drum. Figure 4g–l shows the simulated *Z*(*x*, *y*, ω_d_) between (2,1) and (0,2) modes and the corresponding modal weights are shown as dotted lines in Figure 4m. Similar to the circular drum, the transition of modal weight dominance from (2,1) to (0,2) can also be observed. The effects of the damping parameter to the modal weights are shown in detail in Supporting Information.

#### 2.3.3 Modal Weight Dependence on Driving Frequency

In the observations presented above, we are only able to infer the trend of *e*_*mn*_(ω_d_). To better understand the frequency dependence of *e*_*mn*_(ω_d_), we start with the general equation of motion for a forced clamped plate:^[^ ^24^ ^]^

(3) $$\rho h\frac{\partial^{2}z_{\text{dc+ac}}\left(x,y,t\right)}{\partial t^{2}}+D\nabla^{4}z_{\text{dc+ac}}\left(x,y,t\right)=\frac{\varepsilon_{0}{\left(V_{\text{dc}}+V_{\text{ac}}\cos\left(\omega t\right)\right)}^{2}}{2{\left(g_{0}-z_{dc+ac}\left(x,y,t\right)\right)}^{2}}$$

where *z*_dc + ac_(*x*, *y*, *t*) is the out‐of‐plane displacement, whose Fourier transform is *Z*(*x*, *y*, ω_d_), ρ is the mass density of the plate material, *h* is the plate thickness, and *D* is the flexural rigidity. The right‐hand side of equation (3) is the total driving force per unit area^[^ ^29^, ^30^ ^]^ *F*_dc + ac_(*x*, *y*, *t*), where ε_0_ is the vacuum permittivity, and *g*_0_ is the distance between the drumhead and the bottom electrode. Under the applied force, the total displacement can be presented as a sum of the static and oscillating displacements: *z*_dc + ac_(*x*, *y*, *t*) = *z*_dc_(*x*, *y*) + *z*_ac_(*x*, *y*, *t*). The interferometric detection employed in this work allows the observation of the modal responses to the AC drive. Therefore, we simplify Equation (3) to focus on the AC response, *z*_ac_, to the AC component of the driving force, *F*_ac_. Furthermore, since *V*_dc_ ≫ *V*_ac_, *F*_ac_ can be further simplified by assuming *z*_dc + ac_ to be roughly equal to *z*_dc_ (see Note S2, Supporting Information, for the derivation details). The results of these simplifications will give the following equation of motion:^[^ ^29^, ^31^ ^]^

(4) $$\begin{array}{ccc} \rho h\frac{\partial^{2}z_{\text{ac}}\left(x,y,t\right)}{\partial t^{2}} & + & D\nabla^{4}z_{\text{ac}}\left(x,y,t\right)-\frac{\varepsilon_{0}V_{\text{dc}}^{2}}{{\left(g_{0}-z_{\text{dc}}\left(x,y\right)\right)}^{3}}z_{\text{ac}}\left(x,y,t\right)\\ & = & \frac{\varepsilon_{0}V_{\text{dc}}V_{\text{ac}}\cos\left(\omega t\right)}{{\left(g_{0}-z_{\text{dc}}\left(x,y,t\right)\right)}^{2}} \end{array}$$

The right‐hand side of Equation (4) is now the AC component of the driving force per unit area, *F*_ac_(*x*, *y*, *t*).

**Figure 5:**
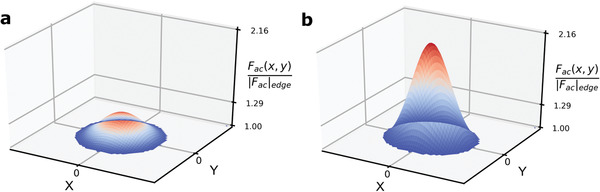
Simulated AC driving force spatial distribution. a,b) Illustrate the driving force spatial distribution for the circular and elliptical drum, respectively. *B*_circle_ = 0.12, which gives maximum *F*_ac_(*x*, *y*)/|*F*_ac_|_edge_ = 1.29, and *B*_ellipse_ = 0.32, which gives maximum *F*_ac_(*x*, *y*)/|*F*_ac_|_edge_ = 2.16.

The next step is to approximate the shape of *z*_dc_. For a clamped circular or elliptical plate under a uniform load, the shape of the deformation can be described by the Bessel function for the (0,1) mode with the following form:^[^ ^32^, ^33^, ^34^, ^35^, ^36^ ^]^

(5) $$z_{\text{dc}}\left(x,y\right)=Bg_{0}{\left(1-\left(\frac{x^{2}}{a^{2}}+\frac{y^{2}}{b^{2}}\right)\right)}^{2}$$

where *a* and *b* are the motional semi‐major and semi‐minor axes for the elliptical drum. In the case of the circular drum, *a* = *b* = *R*, which is the motional radius of the circular drum. The parameter *B* here is the static deformation prefactor that reflects the sharpness of the distribution. Finally, using Equations (1) and (5), *e*_*mn*_(ω_d_) takes the following form (see Note S3, Supporting Information, for the derivation details):

(6) $$e_{mn}\left(\omega_{d}\right)=\frac{\int_{A}Z_{mn}\left(x,y\right)F_{\text{ac}}\left(x,y\right)dx\;dy}{\omega_{mn}^{2}-\omega_{d}^{2}+i\gamma_{mn}\omega_{d}}$$

where γ_*mn*_ is a phenomenological damping parameter for (*m*, *n*) mode, and *F*_ac_(*x*, *y*) is the spatial distribution of the AC driving force amplitude. The time‐dependent driving force has the form: *F*_ac_(*x*, *y*, *t*) = *F*_ac_(*x*, *y*)cos (ω*t*). We take the experimental results as initial values of the damping parameters from their aforementioned *Q* factors, which are 2.39 and 2.35 MHz for γ_21_ and γ_02_ of the circular drum, and 1.30 and 1.99 MHz for γ_21_ and γ_02_ of the elliptical drum, and use *B* as a fitting parameter in Equation (6). The results of the fitting shown in Figures 3 and 4, as previously mentioned, yields *B* = 0.12 for the circular drum and *B* = 0.32 for the elliptical drum, and the final γ_21_ are 2 and 0.3 MHz for the circular and elliptical drum, respectively. With these values of *B*, the respective maximum static deformation at the center for the drums A and B are 35 and 95 nm. These are much larger than the calculated static deformation influenced by *V*_dc_, which are 8.5 and 7.6 pm, respectively (These calculations are derived from the ratio of the maximal resonance amplitude and *Q* factor for a damped driven vibratioPn).^[^ ^2^ ^]^ This implies that there is an initial slack present in the drums even before any electrical actuation. Furthermore, **Figure** **5** shows how the AC force spatial distribution looks like for the drums given their respective values of *B*. The vertical axis is *F*_ac_(*x*, *y*)/|*F*_ac_|_edge_, where |*F*_ac_|_edge_ = ε_0_ *V*_dc_ *V*_ac_/*g*_0_. For the circular drum, the force spatial distribution looks almost uniform across the drumhead. On the other hand, the distribution in the elliptical drum is sharper compared to the circular drum. This difference in sharpness could be explained by the difference in the drums' geometry. To support this, we look at the ratio $\frac{B_{\text{ellipse}}}{B_{\text{circle}}}$ from the experiment and compare it to the theoretically derived expression^[^ ^32^ ^]^ $\frac{\beta_{\text{ellipse}}a^{4}}{\beta_{\text{circle}}R^{4}}$, where β is an eccentricity scaling factor. The ratio from the experimental fit yields 2.67 and is close to 2.54, which is derived from the theoretical expression using *a*, *R*, and β from the reference.^[^ ^32^ ^]^ This difference in sharpness implies that the static deformation of the drums is due to the geometric difference.^[^ ^37^, ^38^ ^]^ In other words, this difference in static deformation translates into a sharper difference in the driving force as $F_{\text{ac}}\approx 1/z_{\text{dc}}^{2}$.

## 3 Discussion

### 3.1 Effect of Driving Force on the Modal Shapes

Equation (6) allows us to infer two significant insights. First, the frequency dependence of the modal weight *e*_*mn*_(ω_d_) is a Lorentzian, which can be interpreted as the frequency response of a damped harmonic oscillator with a resonance frequency ω_*mn*_. Second, *e*_*mn*_(ω_d_) reflects the relation between the force distribution and shape of the (*m*, *n*) mode. This matching is quantified by $\int_{A}Z_{mn}\left(x,y\right)F_{\text{ac}}\left(x,y\right)dxdy$. The force distribution can influence the participation of a particular resonance mode. In both drums, the force distribution is axisymmetric, since the bottom electrode is global. Because of this, the force distribution accentuates the axisymmetric modes such as (0,2) and diminish the participation of non‐axisymmetric modes such as (2,1). This is the reason why, the modal equilibrium points, the points at which the two modes have equal weights, for both drums are closer to the (2,1) mode. In other words, the dominance of the (0,2) mode can be observed in a broader frequency range compared to the (2,1) mode.

From the point of view of drum geometry, we compared the modal equilibrium points of the drums with each other. We see that the equilibrium is closer to the (2,1) mode in the circular drum than the elliptical drum. This means that the additional eccentricity present in the elliptical drum allows the (2,1) mode to widen its dominance in frequency. (The details of the effect of eccentricity on the modal equilibrium position is shown in Supporting Information.) In fact, the presence of the (2,1) mode implies that there is an inherent asymmetry in the system. A perfect circular drum would only show axisymmetric modes.

### 3.2 Off‐Resonance Modal Analysis' Role in Nanomechanical Resonator Studies

This type of analysis can be relevant to coupling of NMRs to other systems.^[^ ^39^ ^]^ First, for intermodal coupling, while it is typical to utilize the fundamental mode, there might be cases where the higher modes are more appropriate to use. One of these works include using multiple electrodes to enhance the driving of the non‐axisymmetric modes and show tunable intermodal coupling.^[^ ^13^ ^]^ Another used parametric excitation to enhance the intermodal coupling.^[^ ^40^ ^]^ Second, the insight obtained from the shape of the spatial distribution of the oscillating component of the force due mainly to an initial slack is highly interesting. Methods of determining initial slack include invasive and destructive means,^[^ ^41^, ^42^, ^43^ ^]^ highly challenging capacitive readout,^[^ ^44^ ^]^ and an optical algorithm that relies on high contrast images from thin layers.^[^ ^45^ ^]^ This analysis provides an alternative means of obtaining initial slack that relies only on normal imaging operation for any geometry of the mechanical oscillator. Furthermore, the analysis could be potentially used to study the damping of mechanical modes as the modal weights are directly influenced by damping as well. Finally and more importantly, this type of analysis is universal. For example, even with a different geometry, the global distribution of the magnetomotive driving force explains the favorability of the detection of the odd modes for the single beam resonators (FEM simulations of a beam geometry with global driving force distribution is shown in Supporting Information).^[^ ^46^ ^]^ The wealth of potential studies involving coupled NMRs makes the knowledge gained from the effect of the driving force on the resonance modes an invaluable tool.

## 4 Conclusion

In summary, we observed intermodal transitions of NbSe_2_ mechanical plate drums through spatial mapping at off‐resonance frequencies. We, then, described the off‐resonance motion as a superposition of the resonance modes. Through this modal analysis, we were able to see how participation of resonance modes changes as the driving frequency changes. Furthermore, by looking at the modal weight formula, we were able to describe how the modal shapes are revealed through the application of the driving force and deduce how this driving force is distributed across the oscillator. In fact, all mechanical vibrations of objects are mixtures of their resonance modes across the driving frequency spectrum with varying weights of participation. This fundamental and universal understanding of the relationship between the resonance modes and the driving force will greatly benefit all future NMRs studies, which will inevitably involve coupling of the flexural motion to various degrees of freedom of different nature and energy.

## 5 Experimental Section

##### Sample Fabrication

40 nm Au and 20 nm Cr electrodes were lithographically patterned on 7 mm × 7 mm × 0.65 mm p‐doped Si chips with a thermally grown 543 nm thick SiO_2_ layer. The chip was then cleaned through ultrasonication for 10 min in acetone, 2 min in IPA, and 1 min in DI water. The drums' spacer was then created by spin coating the chip with the AR‐P (CSAR‐62) electron beam resist. After baking at 180 °C for 1 min, the resist was patterned with the drum hole and contact window patterns. After development, the resist was baked again at 180 °C for 9 min to make it rigid. The spacer thickness was measured to be 295 ± 10 nm using a commercial stylus profilometer. Bulk NbSe_2_ purchased from HQ Graphene were exfoliated and transferred onto the patterned drum and contact windows using a deterministic dry PDMS stamp transfer process.^[^ ^47^, ^48^ ^]^

##### Mechanical Detection and Actuation

A continuous‐wave green laser beam (532 nm), with a power of 800 µW and beam diameter of about 1.8 µm, was focused on the drumhead and the intensity modulation caused by the interfering reflections from the semi‐transparent flake, NbSe_2_ in this case, and the gold electrode underneath was captured by an avalanche photodetector. A scanning mirror was used to move the laser spot across the drumhead thereby making it possible to spatially resolve the recorded driven response. The driving of the mechanical drums was done using the electromotive scheme by supplying a voltage from a function generator with an oscillating component (*V*_ac_) at 250 mVpp with a frequency close to the mechanical resonance frequency of the drum, and a dc component (*V*_dc_) at 4 V. The dc component of the applied voltage allowed amplification of the ac component, which improved the signal‐to‐noise ratio without the need of strong drive that might result in the non‐linear regime of the resonator.^[^ ^39^ ^]^ For clarity, all measurements were done in the linear regime. A lock‐in amplifier recorded the signal from the avalanche photodetector with a reference signal coming from the function generator. All measurements were done at room temperature (≈ 25 °C) with a vacuum box pressure of about 10^−7^ mbar.

##### Statistical Analysis—Experimental Modal Weight Data

For the preparation of the data, the following were the steps done to obtain the experimental modal weights *e*_*mn*_:

1. Get normalization factor (NF):
  a) square all elements of [*Z*];
  b) add all elements from (a);
  c) NF = square root the result of (b).
2. Get modal weight *e*_*mn*_ for off resonant mode ω_*d*_:
  a) multiply by element of $\left[Z_{mn}\right]$ and $[Z_{\omega_{d}}]:$
  b) *e*_*mn*_ = sum all elements of from (a).

The estimated error of experimental data points from Figures 3m and 4m is about 0.07% through error propagation.

##### Statistical Analysis—FEM Simulations

FEM simulations were done using COMSOL. The details of the study are as follows:

1) A first study of frequency modal prestressed analysis is done with the following steps:
  a. Stationary step that calculates the prestress deformation due to *V*_dc_;
  b. Eigenfrequency step that calculates the eigenfrequencies and corresponding mode shapes based on geometry;
  c. Frequency domain, modal step that sweeps the driving frequency to get the modal shapes.
2) A second parametric study of mapping and calculation that uses the results from Study 1 to retrieve the modal weights. The projection of the resulting eigenmode shapes of Study 1b is calculated from all the driven modal shapes of Study 1c.

The thickness is used as a parameter in the simulations to match the observed resonant frequencies.

## Conflict of Interest

The authors declare no conflict of interest.

## Author Contributions

C.D.C. conceived the device and supervised the project; J.C.E. fabricated the samples; K.‐H.L. and C.‐Y.Y. designed and built the setup for optical measurements; J.C.E., M.A.C.A., and C.‐Y.Y performed the measurements; J.C.E., M.A.C.A., J.Y.W., S.K., Y.P., and C.D.C analyzed the data, performed simulations, and wrote the manuscript; all authors discussed the results and contributed to the manuscript.

## Supporting information

Supporting Information

## Data Availability

Research data are not shared.
