# Supplementary material for: Imaging Off‐Resonance Nanomechanical Motion as Modal Superposition
Source: Adv Sci (Weinh). 2021 May 19;8(13):2005041. doi: 10.1002/advs.202005041 (PMC8261521; doi:10.1002/advs.202005041)
Supplement: Supplementary file 1 — Supporting Information [file ADVS-8-2005041-s001.pdf]

© 2021 Wiley-VCH GmbH

ADVANCED SCIENCE
Open Access

Supporting Information

for *Adv. Sci.,* DOI: 10.1002/advs.202005041

Imaging off-resonance nanomechanical motion as modal superposition

JoshouaCondicion Esmenda, Myrron Albert Callera Aguila, Jyh-Yang Wang, Teik-Hui Lee, Chi-Yuan Yang, Kung-Hsuan Lin, Kuei-Shu Chang-Liao, Nadav Katz, Sergey Kafanov, Yuri Pashkin, and ChiiDong Chen

Supplementary Information for

Imaging off-resonance nanomechanical motion as modal superposition

JoshouaCondicion Esmenda,[1,2,3]Myrron Albert Callera Aguila,[1,2,3,]Jyh-Yang Wang,[3]Teik-Hui Lee,[3]Chi-Yuan Yang,[3,4]Kung-Hsuan Lin,[3]Kuei-Shu Chang-Liao,[1]Nadav Katz,[5]Sergey Kafanov,[6]Yuri Pashkin,[6]and ChiiDong Chen[3]

[1)]*Department of Engineering and System Science, National Tsing Hua University, Hsinchu 30013, Taiwan*

[2)]*Nano-Science and Technology Program, Taiwan International Graduate Program, Academia Sinica, Taipei 11529, Taiwan*

[3)]*Institute of Physics, Academia Sinica, Taipei 11529, Taiwan*

[4)]*Department of Physics, National Taiwan University, Taipei 10617, Taiwan*

[5)]*Racah Institute of Physics, Hebrew University, Jerusalem, 91904 Israel*

[6)]*Department of Physics, Lancaster University, LA1 4YB, Lancaster, United Kingdom*

Supplementary Note 1. Dry deterministic transfer

Bulk two-dimensional materials, in this case, NbSe2, are exfoliated by using 3% polydimethylsiloxane (PDMS) cured on flexible and transparent commercially available cellulose acetate sheets (CAS), also commonly known as transparencies. The bulk is mechanically exfoliated similar to the Scotch tape method but we opted to use PDMS to control the viscosity (stickiness) for our purposes. When the desired flake thickness (estimated from its optical transparency) is achieved, the target flake is then transferred onto a less viscous 9% PDMS cured on an ordinary microscope slide. Theslidewith the target flake is then mounted onto a microscope stage. The target chip is then placed onto another stamping stage where the chip and the target flake can be aligned independently, while a live feedthrough the objective lens underneath is used to monitor the stamping process[1,2]. Through this dry deterministic transfer, we are able to avoid exposing the 2D material to any wet solvents that might compromise the material's unique mechanical properties. All the fabricationsteps are shown in Figure S1.

Supplementary Note 2. Equation governing the mechanical vibration under DC and AC voltage

For an electrostatically actuated circular plate, the out-of-plane (z direction) displacement from equilibrium, which includes the static part $z_{dc}(x,y)$and the time dependent part $z_{ac}(x,y,t)$, is describedby the following equation [3]:

$$\rho h\frac{\partial^2 z_{dc+ac}(x,y,t)}{\partial t^2} + D\nabla^4\left[z_{dc}(x,y)+z_{ac}(x,y,t)\right] = \frac{\varepsilon_0\left[V_{dc}+V_{ac}(t)\right]^2}{2\left[g_0 - z_{dc}(x,y) - z_{ac}(x,y,t)\right]^2},(1)$$

where ρ is the mass density of $NbSe_2$, h is the flake thickness, D is the flexural rigidity and g_0 is the distance between the drumhead and the bottom electrode at $V_{dc}=0$. The term on the right-hand side is the electrostatic force per unit area due to the applied voltage$V_{dc}+V_{ac}(t)$ and $z_{dc}(x,y)$satisfies the following equation:

$$D\nabla^4 z_{dc}(x,y) = \frac{\varepsilon_0 {V_{dc}}^2}{2\left[g_0 - z_{dc}(x,y)\right]^2}.(2)$$

To obtain the equation describingthe mechanical vibration due to $V_{ac}(t)$, we expand$\left[g_0 - z_{dc}(x,y) - z_{ac}(x,y,t)\right]^2$ in the force term at $g_0 - z_{dc}(x,y)$ usingTaylor series. Accordingly, Eq. (1) can be re-written as follows:

$$\rho h\frac{\partial^2 z_{ac}}{\partial t^2} + D\nabla^4 z_{dc} + D\nabla^4 z_{ac} = \frac{\varepsilon_0\left[V_{dc}+V_{ac}(t)\right]^2}{2}\left[\frac{1}{(g_0 - z_{dc})^2} + \frac{2z_{ac}}{(g_0 - z_{dc})^3} + \frac{3{z_{ac}}^2}{(g_0 - z_{dc})^4} + \cdots\right].(3)$$

Under the condition of $V_{dc} \gg V_{ac}(t)$and $z_{dc} \gg z_{ac}$, the first order approximation of Eq. (3) is given below:

$$\rho h \frac{\partial^2 z_{ac}(x,y,t)}{\partial t^2} + D\nabla^4 z_{ac}(x,y,t) - \frac{\varepsilon_0 {V_{dc}}^2}{[g_0 - z_{dc}(x,y)]^3} z_{ac}(x,y,t) = \frac{\varepsilon_0 V_{dc}}{[g_0 - z_{dc}(x,y)]^2} V_{ac}(t) \quad ,(4)$$

which is the equation describing $z_{ac}(x,y,t)$ under the AC driving voltage$V_{ac}(t)$. The third term on the left-hand side of Eq. (4) is responsible for the additional change of mechanical properties of thepre-stressed plate due to V_{dc}. It is also noted that the AC force per unit area, $F_{ac}(x,y,t) = \frac{\varepsilon_0 V_{dc} V_{ac}}{[g_0 - z_{dc}(x,y)]^2}$, is dependent on the static displacement$z_{dc}(x,y)$. Provided that$V_{ac}(t) = V_{ac}\cos(\omega_d t)$, $F_{ac}(x,y,t)$isalso time dependentof the form $F_{ac}(x,y)\cos(\omega_d t)$, where $F_{ac}(x,y)$is the force distribution given as:

$$F_{ac}(x,y) = \frac{\varepsilon_0 V_{dc} V_{ac}}{[g_0 - z_{dc}(x,y)]^2}. \qquad (5)$$

Supplementary Note 3. Normal Mode Expansion

To investigate the frequency response of $z_{ac}(x,y,t)$ for $F_{ac}(x,y,t) = F_{ac}(x,y)\cos(\omega_d t)$, we considerthe following expansion for the stationary solution of $z_{ac}(x,y,t)$:

$$z_{ac}(x,y,t) = \sum_{k=0}^{\infty} Z_k(x,y) e_k(t) \quad ,(6)$$

where $Z_k(x,y)$are complex amplitudes of spatial natural modes, or normal modes, of vibration and $e_k(t)$ are the corresponding dimensionless time-dependent expanding coefficients. It is noted that $Z_k(x,y)$ form a basis set, which satisfies the following equation overthe drum area *A* and the boundary conditions at boundary *C*.

$$D\nabla^4 Z_k(x,y) - \frac{\varepsilon_0 V_{dc}^2}{(g_0 - z_{dc})^3} Z_k(x,y) = \rho h \omega_k^2 Z_k(x,y), \qquad x,y \in A \quad , (7)$$

$$Z_k(x,y) = 0, \qquad x,y \in C \quad , (8)$$

$$\nabla_{\hat{n}} Z_k(x,y) = 0, \qquad x,y \in C \quad ,(9)$$

where ω_k is the eigenfreqency of eigenmode $Z_k(x,y)$and $\hat{n}$is the direction normal to boundary *C*. To obtain anequation for$e_k(t)$, we multiply both sides of Eq. (4) by$Z_k^*(x,y)$ and integrate over the drum area. As a result, we have:

$$\int_A Z_k^*(x,y)\left[\rho h \frac{\partial^2 z_{ac}(x,y,t)}{\partial t^2} + D\nabla^4 z_{ac}(x,y,t) - \frac{\varepsilon_0 V_{dc}^2}{(g_0 - z_{dc})^3} z_{ac}(x,y,t)\right] da = \cos(\omega_d t) \int_A Z_k^*(x,y) F_{ac}(x,y) da \quad (10)$$

Plugging Eqs. (6) and (7) into Eq. (10) and applying the orthogonal condition for $Z_k(x,y)$, we obtain the equation for $e_k(t)$ as follows:

$$\frac{d^2}{dt^2} e_k(t) + \gamma_k \frac{d}{dt} e_k(t) + \omega_k^2 e_k(t) = \frac{\frac{1}{\rho h}\int_A Z_k^*(x,y) F_{ac}(x,y) da}{\int_A |Z_k(x,y)|^2 da} \cos(\omega_d t), \quad (11)$$

in which we have introduced a phenomenological damping term γ_k. In describing the frequency response of $e_k(t)$, we use the phasor $e_k(\omega_d)$, which is defined as $e_k(t) = Re\left(e_k(\omega_d) e^{i\omega_d t}\right)$. The solution of $e_k(\omega_d)$ is given as:

$$e_k(\omega_d) = \frac{1}{\omega_k^2 - \omega_d^2 + i\gamma_k \omega_d} \frac{\frac{1}{\rho h}\int_A Z_k^*(x,y) F_{ac}(x,y) da}{\int_A |Z_k(x,y)|^2 da}. \quad (12)$$

Accordingly, the frequency response of $z_{ac}(x,y,t)$ is

$$z_{ac}(x,y,\omega_d) = \sum_{k=0}^{\infty} Z_k(x,y) e_k(\omega_d), \quad (13)$$

where $Z_k(x,y)$ is normalized such that the value of $\int_A |Z_k(x,y)|^2 da$ is unity with the dimensionality of the fourth power of length.

Supplementary Note 4. Matrix form analysis of normal mode expansion

The equation of motion for an undamped mechanical oscillator system having multiple degrees of freedom is given by:

$$[\mathbf{M}]\left\{\ddot{Z}(t)\right\} + [\mathbf{K}]\{Z(t)\} = \{f(t)\}, \quad (14)$$

where $[\mathbf{M}]$ is the mass matrix, $[\mathbf{K}]$ is the stiffness matrix, {Z(t)} is the vector of displacement from equilibrium and $\{f(t)\}$ is the excitation force vector. For modal analysis, the mass-normalized mode shapes are more useful because they are unique presentations of the mode shapes. The mass-normalized mode shapes satisfy the following equations:

$$[\Phi]^T[\mathbf{M}][\Phi] = [\mathbf{I}], \quad (15)$$

$$[\Phi]^T[\mathbf{K}][\Phi] = \left[\omega_k^2\right], \quad (16)$$

where $[\Phi]$ is the mass-normalized mode shape matrix, $[\Phi]^T$ is the transposed mass-normalized mode shape matrix,and $[I]$ is the identity matrix.If the harmonic excitation force has the frequency close to the resonance frequency and zero phase shift, it can be presented as:

$$\{f(t)\} = \{F\}\sin\omega_d t. \quad (17)$$

Then the response of the system to the external drive is also harmonic:

$$\{Z(t)\} = \{Z\}\sin\omega_d t. \quad (18)$$

Substituting equations (17) and (18) into (14), we arrive atthe following equation:

$$\{Z(t)\} = [\Phi][\omega_k^2 - \omega_d{}^2]^{-1}[\Phi]^T\{F\}\sin\omega_d t. \quad (19)$$

Supplementary Note 5. COMSOL Simulations for the MO devices

Frequency Modal Analysis feature of the COMSOL simulator was used to analyze the normal modes of devices A and B. Both deviceshave almost the same parameters except for their eccentricity, which is the ratio of the major and minor axes. For device B, the eccentricity is equal to 1.140. For device A, an eccentricity of 1.041 is needed to approximate the response shapes of the measureddata. This implies that there is actually a small asymmetry in device A even though it was designed to be circular. This supports the idea that a perfect symmetry in actual devices is almost impossible to achieve. Furthermore, we had to introduce negative values of the initial strain, indicative of a loose clamping of the drums. Figures S2 and S3 show the normal mode shapes and corresponding frequencies of devices A and B, respectively.Figure S4 shows the modal weight percentage for both drums in a wider driving frequency range for all six lowest modes using the matrix method described in Supplementary note 4.To analyze this figure, we could associate the existence or influence of a resonance mode with its frequency range where its modal weight is nonzero, or modal weight bandwidth for short. For example, looking at the fundamental mode for both devices, its modal weight bandwidth is one of the largest among the lowest six modes. If we compare this to the (1,1) modes, their modal weight bandwidth cannot be seen by the simulation results because it is simply too sharp. In other words, it appears that the system favors the fundamental mode significantly more than the (1,1) modes because the modal weight bandwidth for the fundamental mode is larger than the (1,1) modes.This concept of modal weight bandwidth will aid us in analyzing the modal weight behavior of each resonance mode.

At this point, we must also take note of a simulation limitation. We will refer to section 5.3.2 FEM Simulations of the main article. The results the eigenfrequency of Step 1)b gives a value with three decimal places for practicality. This is sufficient enough for favorable modes such as the fundamental mode but is not for unfavored modes such as the (1,1) modes. The exact

calculated eigenfrequency for the all modes could have more decimal points. However, if the mode is unfavored, its modal weight bandwidth is so small that even the eigenfrequency result from Step 1)b is already not 100%. This should not be interpreted as a contradiction to the orthogonality of the resonance modes, where we expect 100 % modal weight value at its resonance frequency (if no damping considered).

Furthermore, to fully explore the effect of eccentricity to the modal weight, simulations were made by varying the eccentricity of the drum. The modal weights between (2,1) and (0,2) modes were retrieved and the modal equilibrium point frequency is determined from these. Figure S5 shows drum eccentricity versus the modal equilibrium point frequency. It shows that the modal equilibrium point frequency shifts towards (0,2) frequency as the eccentricity is increased. This supports the idea that eccentricity could affect modal participation.

Supplementary Note 6. Frequency Modal Analysis for a beam structure

As a further support for our claim that intermodal coupling, as explained by the normal mode expansion, is a universal phenomenon, we have performedanother COMSOL simulation with a different type of a mechanical resonator, namely a doubly clamped beam structure with a global actuating electrode. Figure S6 shows the results of this simulation and similar to theanalysis of our experiment, the normal modes do participate withvarying weights throughout the driving frequency range. Using the concept of the modal weight bandwidth, we could see thatodd modes, 1, 3, and 5 are dominating, and the even modes are not. This is due to the global actuating electrode that implies a global distribution of the actuation force.

Supplementary Note 7. The effect of varying the damping parameter to the modal weight

Figure S7 illustrates the effect of the damping parameterγ to the modal weight$e$ of device A as an example. As the damping parameter of a resonant mode becomes smaller (or the Q value getting higher) the modal weight of that resonant mode becomes closer to 100% at its resonant frequency. Furthermore, this effect is more apparent for those modes that are not enhanced by the actuating electrode, like the (2,1) mode in this case. On the other hand, this effect is not clear for the (0,2) mode.

Supplementary Note 8. The effect of thickness to the modal weight

The following equation motion is for a tensioned plate [4]:

$$\nabla^2(z) - \frac{T}{D}\nabla^2(z) + \frac{\rho}{D}\frac{\delta^2 z}{\delta t^2} = 0 \tag{20}$$

The resonance frequency for the *(m,n)* mode is given by: [5]:

$$f_{mn} = \sqrt{\left(f_{mn}^{bending}\right)^2 + \left(f_{mn}^{stretching}\right)^2} = \sqrt{\left(f_{mn}^{plate}\right)^2 + \left(f_{mn}^{membrane}\right)^2} \tag{21}$$

The following are the respective equations for membrane and plate resonance frequencies [6]:

$$f_{mn}^{membrane} = \sqrt{\left(\frac{\lambda_{mn}^{membrane}}{2\pi R}\right)^2 \frac{\sigma}{\rho}} \tag{22}$$

$$f_{mn}^{plate} = \sqrt{\left(\frac{\lambda_{mn}^{plate^2}}{2\pi R^2}\right)^2 \frac{D}{\rho h}} \quad (23)$$

where σ is the tensile stress, and λ are the respective eigenmode numbers for membrane and plate resonances. The tensile stress can be further described in terms of the total radial tension T to counteract the F_{DC}:

$$\sigma = \frac{T}{2\pi R h} = \frac{C'_g V_{dc}^2}{4\pi R h} \quad (24)$$

$$C'_g = \frac{\varepsilon_0 A}{g_0^2} \quad (25)$$

So the final equation for the frequency of a tensioned plate is:

$$f_{mn} = \sqrt{\left(\frac{\lambda_{mn}^{plate^2}}{2\pi R^2}\right)^2 \frac{D}{\rho h} + \left(\frac{\lambda_{mn}^{membrane}}{2\pi R}\right)^2 \frac{C'_g V_{dc}^2}{4\pi R h \rho}} \quad (26)$$

On the other hand, the modal weight is given by:

$$e_{mn}(f_d) = \frac{\int_A \mathbf{Z}_{mn}(x,y) F_{ac}(x,y)\,dx\,dy}{f_{mn}^2 - f_d^2 + i\gamma_{mn} f_d} \quad (27)$$

In this equation, only f_{mn} is influenced by the thickness. This means only the spacing between modes are changed but the modal weight trend between these resonances will not. The only factors that can change the modal weight behavior are 1) Fac, which is explored in the discussion of the main article, 2) $\mathbb{Z}_{mn}$, which is briefly explored in Supplementary Note 5 as the effect of eccentricity change, and 3) γ_{mn}, which is analyzed in Supplementary Note 7.

Supplementary Note 9. V_{dc} dependence of resonance amplitude and frequency

From Supplementary Note 8, we could see the dependence of the resonance frequency to V_{dc}.Figures S9 and S10 show the V_{dc} graphs for devices A and B, respectively. From Figure S9b and S10b, we could see that there is almost zero significant change to resonant frequency as V_{dc} is changed. This is because at total radial tension induced by V_{dc} is not significant enough compared to the flexural rigidity of the devices. Tension = 3.13 x10^{-8} N while D = 2.99 x 10^{-5} Nm. In other words, the devices are dominated by bending significantly more than the stretching induced by V_{dc}.

On the other hand, the static deformation dependence to $V_{dc}$is shown in Figures S9d and S10d as extracted from the Q factor and maximum motional amplitude [6].

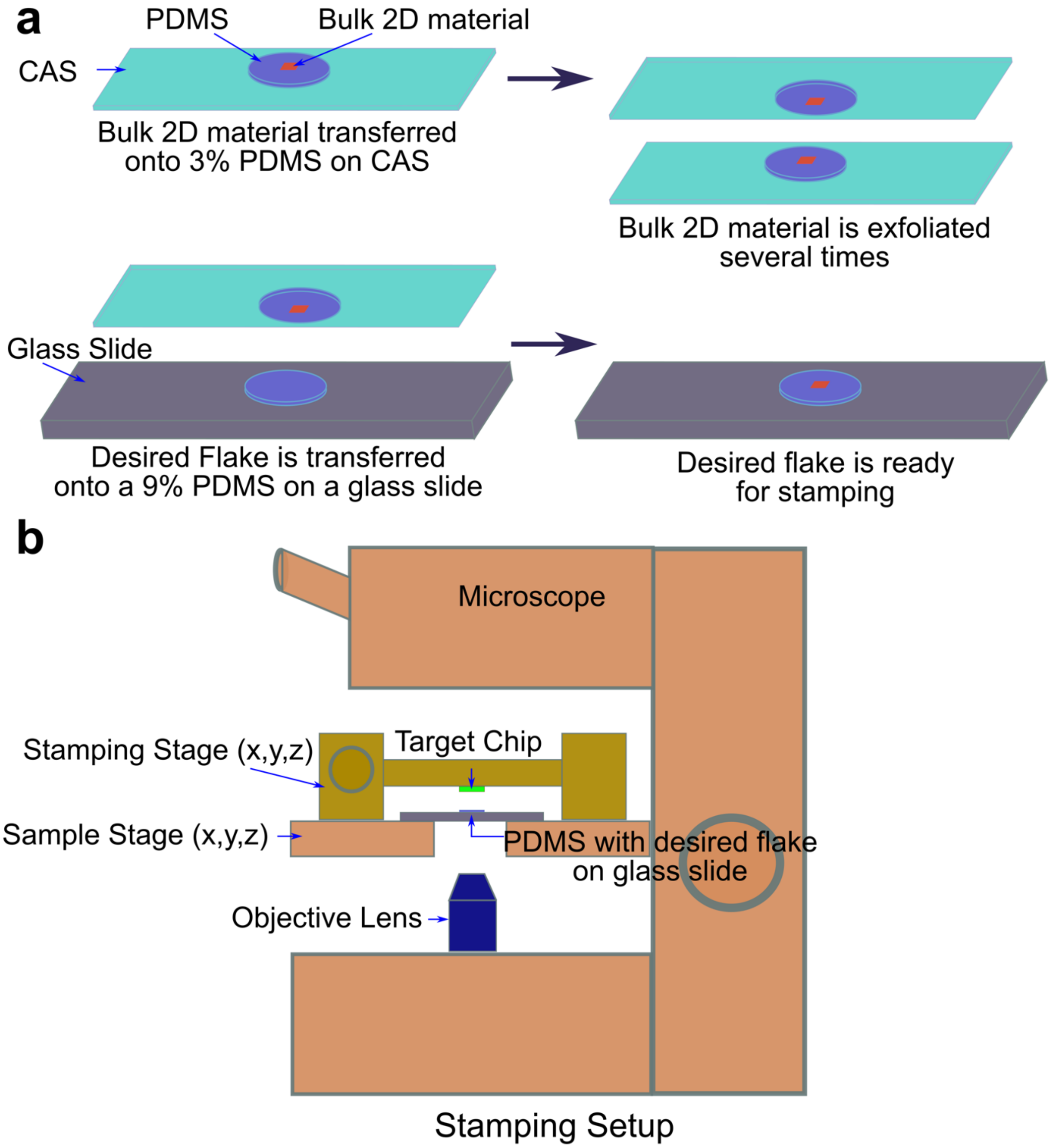

Figure S1. Dry deterministic transfer.

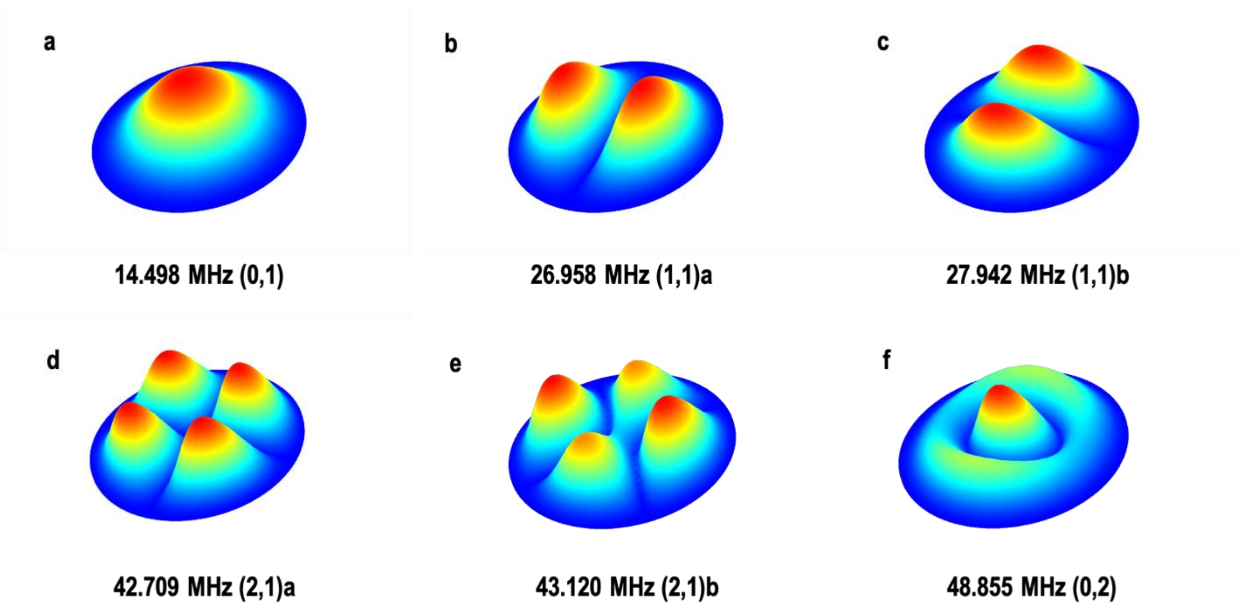

Figure S2. First six resonant modes (a-f) for Device A.

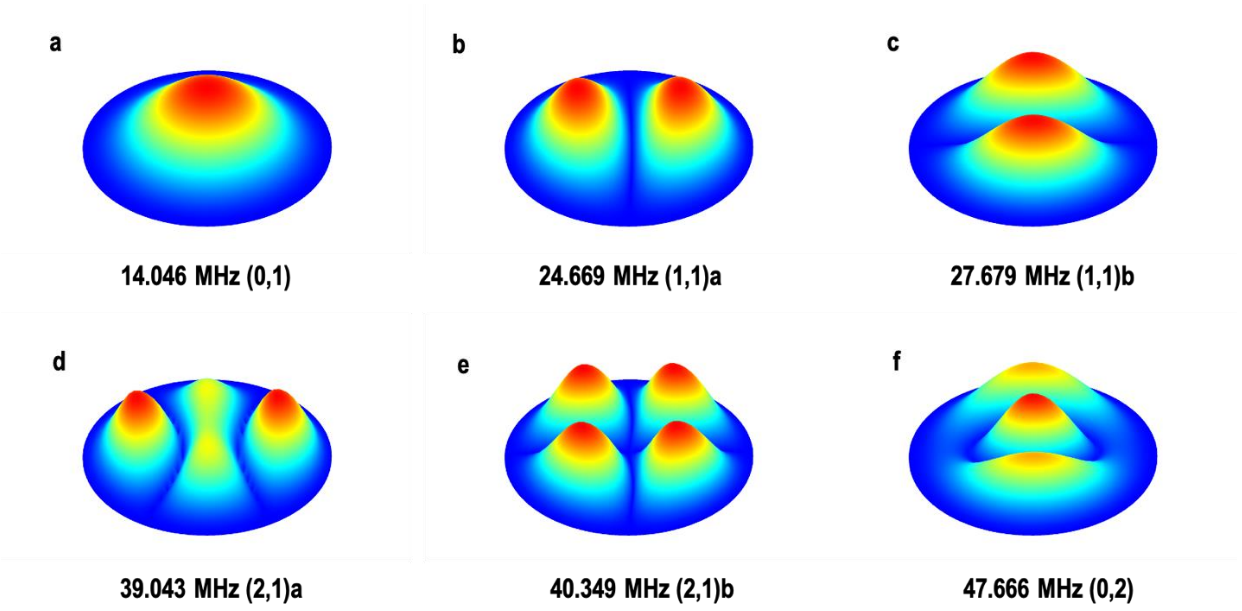

Figure S3. First six resonant modes (a-f) for Device B.

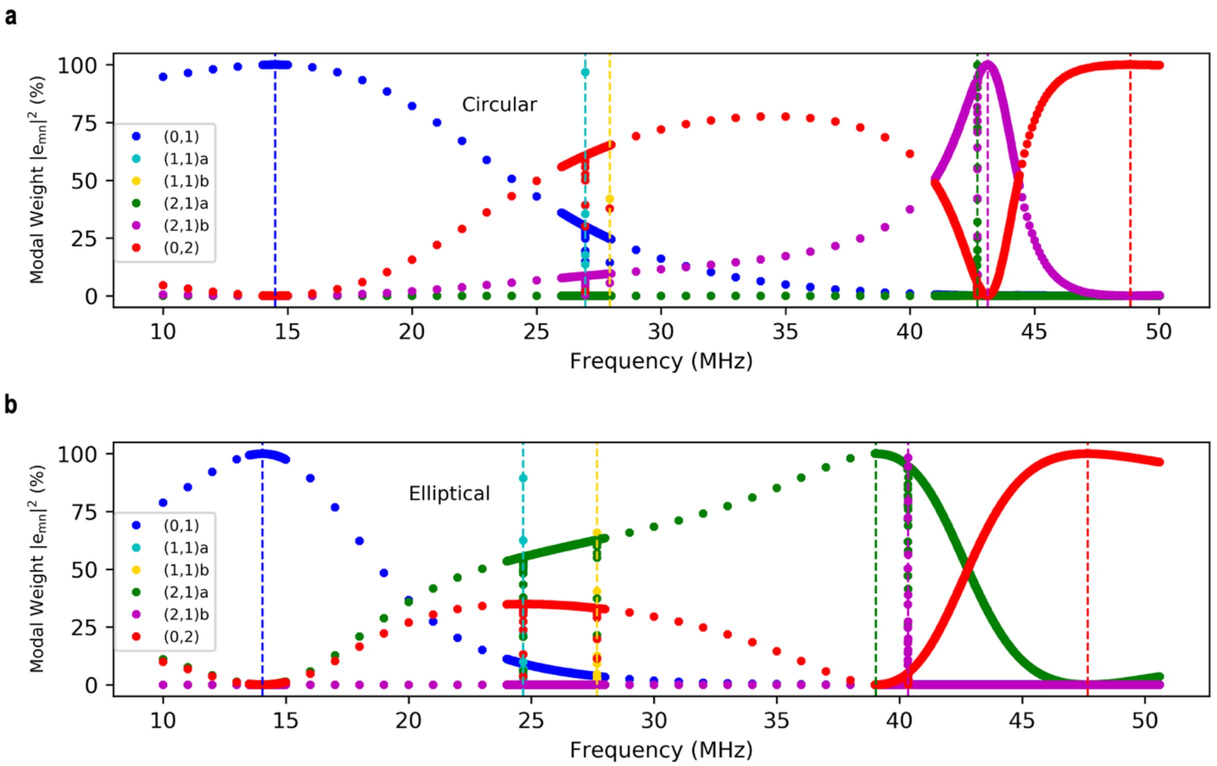

Figure S4. Modal weight percentage for the a) circular and b) elliptical drums in a wider range of the driving frequency for all six lowest modes. The dashed vertical lines indicate the corresponding resonance frequencies.

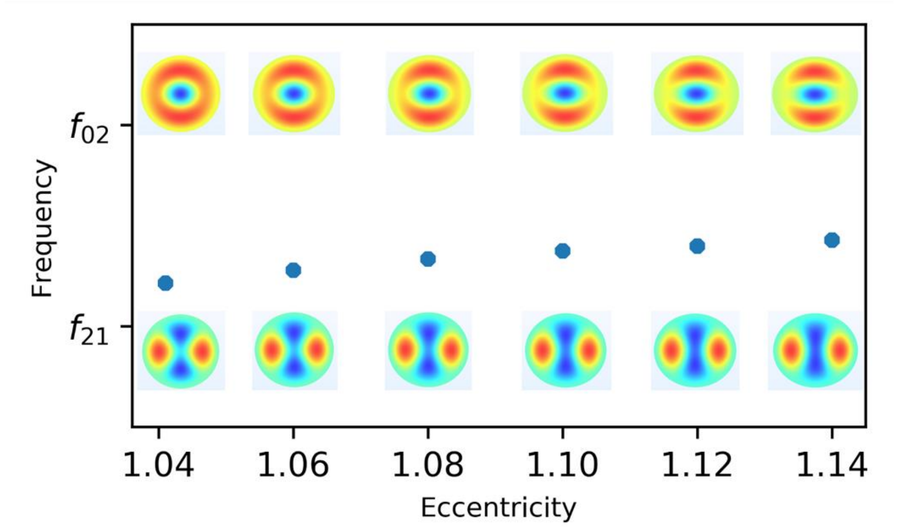

Figure S5. Frequency of equal modal weights versus eccentricity for modes (2,1) and (0,2).

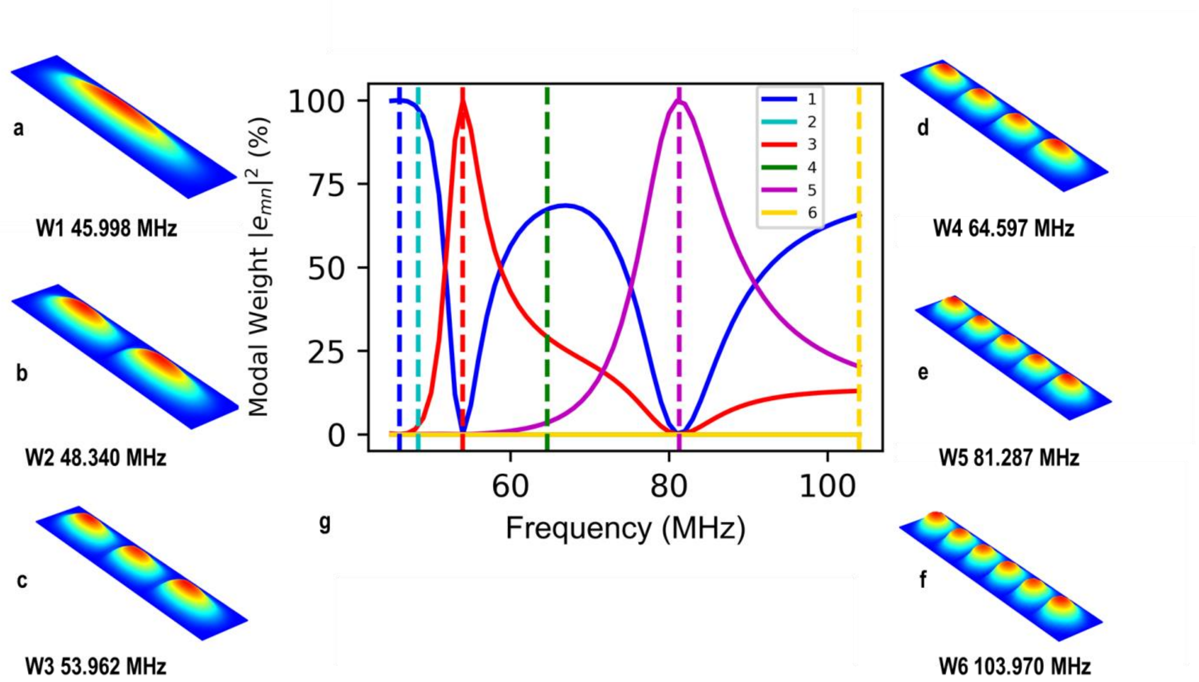

Figure S6. Resonant modes for a doubly clamped beam structure and corresponding frequency modal analysis. (a) – (f) depict resonance modes starting from the fundamental (a) up to the sixth mode (f). (g) shows the frequency dependence of the modal weight for all six modes. The dashed vertical lines indicate the resonance frequencies of the normal modes.

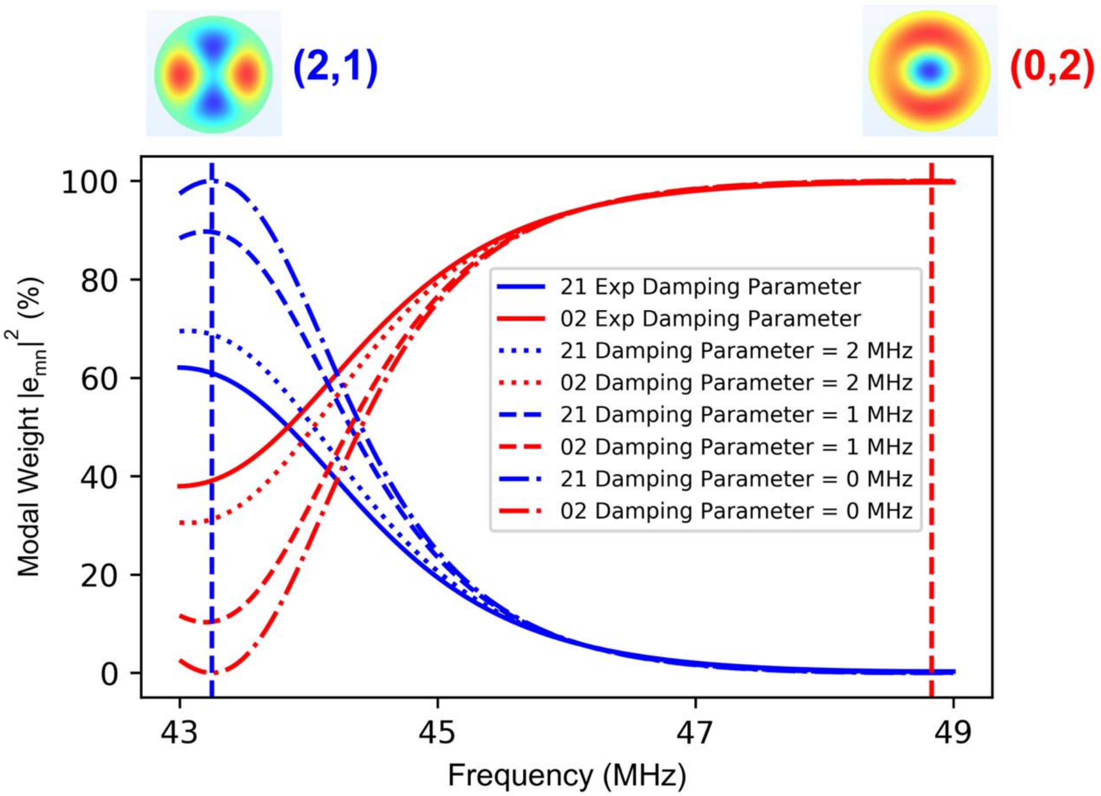

Figure S7. Effect of varying damping parameter to the modal weight.

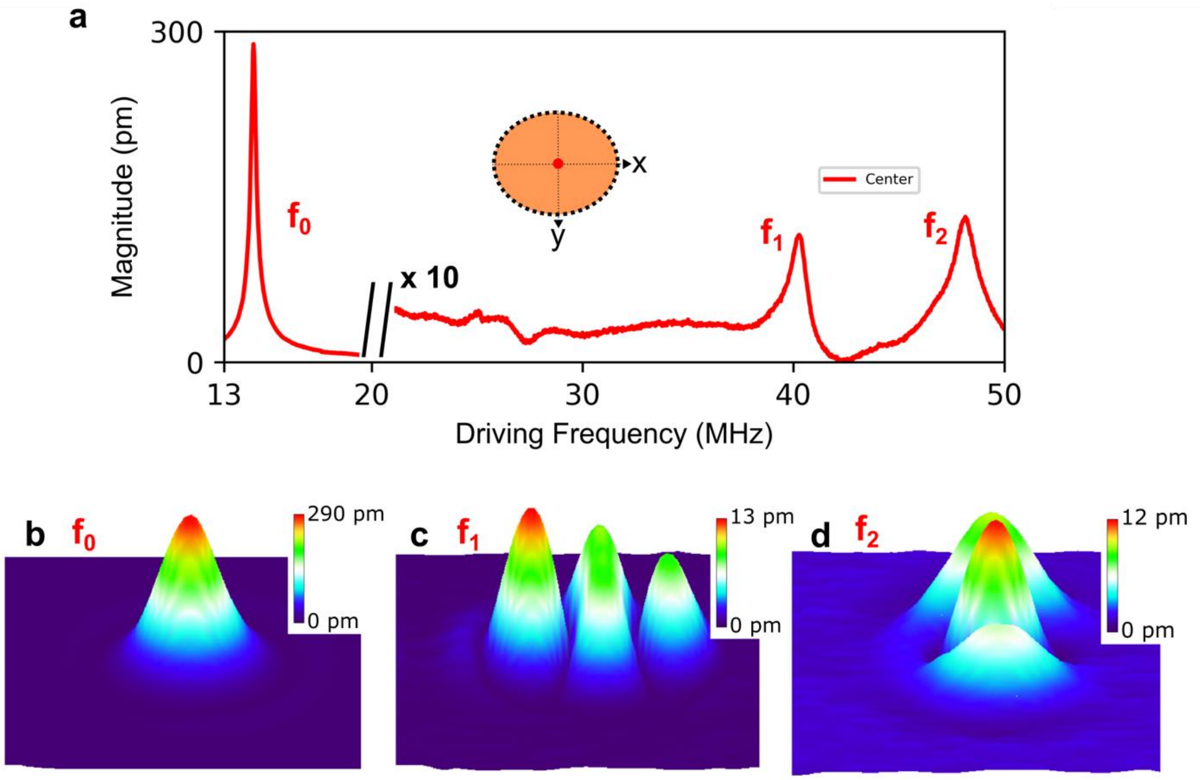

Figure S8. Resonant mode mapping for device B. (a) Response spectrum retrieved for device B at the center for the drum. Similar to device A, magnitude of the spectrum is scaled to 10x beyond 20 MHz. Three peaks are identified in the spectrum: f_0 at 14.4 MHz, f_1 at 40.28 MHz, and f_2 at 48.15MHz. (b) to (d) are the corresponding mode mapping of the aforementioned peaks.

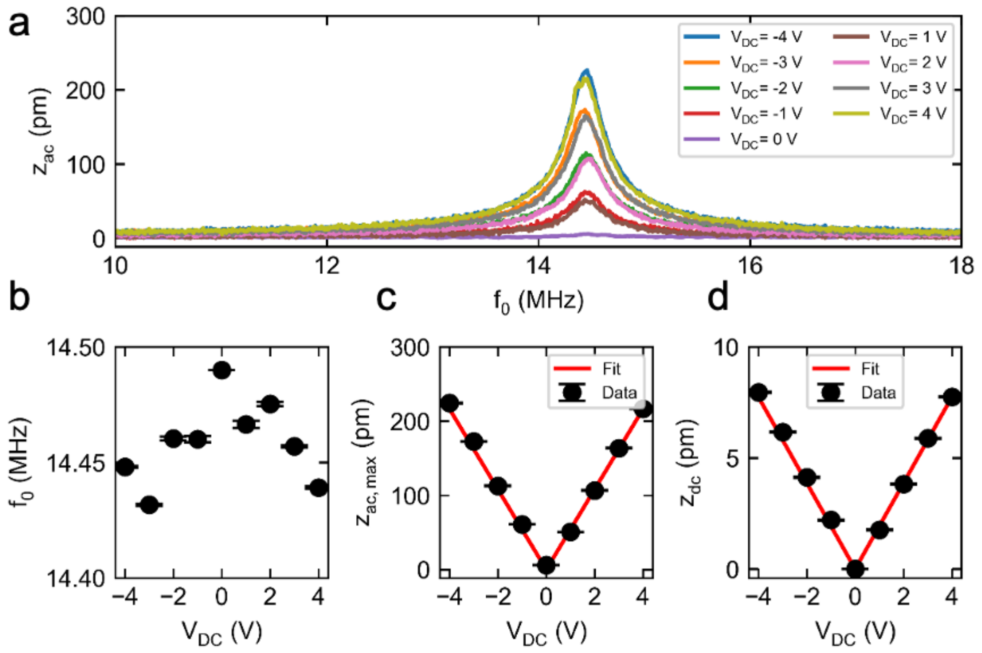

Figure S9. V_{dc} dependence of device A. (a) Multiplot of V_{dc} dependence of the fundamental mode of device A. (b) Frequency dependence. (c) Motional amplitude dependence. (d) Static deformation dependence.All curves are retrieved with V_{ac} = 250 mVpp.

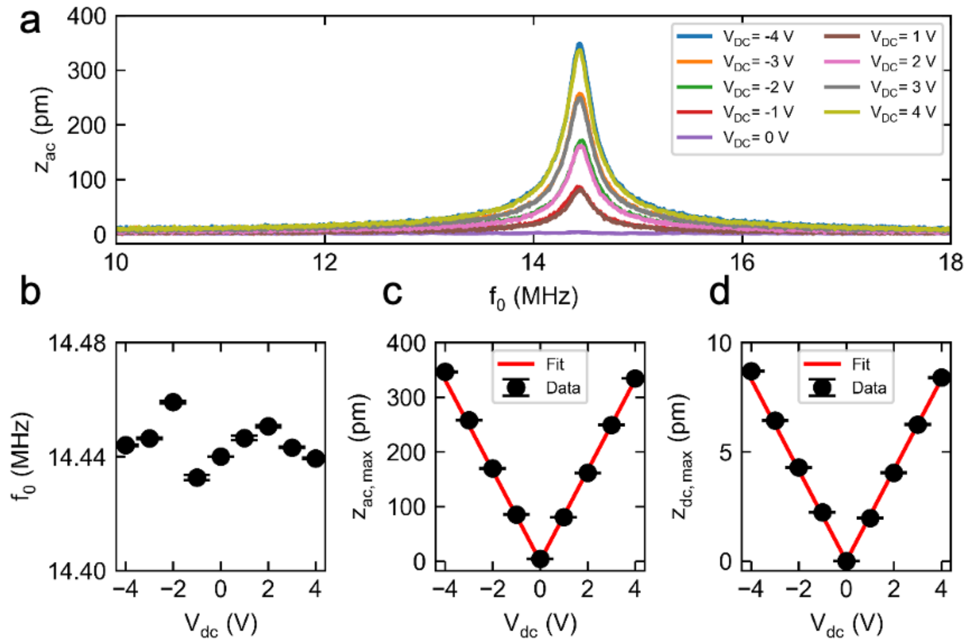

Figure S10. V_{dc} dependence of device B. (a) Multiplot of V_{dc} dependence of the fundamental mode of device B. (b) Frequency dependence. (c) Motional amplitude dependence. (d) Static deformation dependence. All curves are retrieved with V_{ac} = 250 mVpp.

References:

[1] A. Castellanos-Gomez, M. Buscema, R. Molenaar, V. Singh, L. Janssen, H. S. van der Zant, and G. A.Steele, “Deterministic transfer of two-dimensional materials by all-dry viscoelastic stamping,” 2D Materials1, 011002 (2014).

[2] G. Pande, J.-Y. Siao, W.-L. Chen, C.-J. Lee, R. Sankar, Y.-M. Chang, C.-D. Chen, W.-H. Chang, F.-C.Chou, and M.-T. Lin, “Ultralow Schottky barriers in hexagonal boron nitride-encapsulated monolayer WSe2 tunnel field-effect transistors,” ACS Applied Materials & Interfaces12, 18667–18673 (2020).

[3] Q.-A. Huang,“Micro electro mechanical systems,”Springer, (2018).

[4] T Wah,“Vibration of circular plates,” the Journal of the Acoustical Society of America,(1962).

[5] Vera A.Sazonova, "A tunable carbon nanotube resonator." (2006).

[6] Schmid, Silvan, Luis Guillermo Villanueva, and Michael Lee Roukes,“Fundamentals of nanomechanical resonators,” Vol. 49. Berlin: Springer, (2016).
